# Retraction Note: Chondro-protective effects of polydatin in osteoarthritis through its effect on restoring dysregulated autophagy via modulating MAPK, and PI3K/Akt signaling pathways

**DOI:** 10.1038/s41598-020-68880-9

**Published:** 2020-07-13

**Authors:** Zhengyuan Wu, Zhiwei Luan, Xiaohan Zhang, Kai Zou, Shiting Ma, Zhenyi Yang, Wenyu Feng, Mingwei He, Linhua Jiang, Jia Li, Jun Yao

**Affiliations:** 1 Department of Orthopaedics Trauma and Hand Surgery, The First Affiliated Hospital of Guangxi Medical University, Nanning, 530021 China; 2 Department of Bone and Joint Surgery, The First Affiliated Hospital of Guangxi Medical University, Nanning, 530021 China; 3 Departments of Pathology, The First Affliated Hospital of Guangxi Medical University, Nanning, 530021 China; 4 Guangxi Collaborative Innovation Center for Biomedicine, Life Sciences Institute, Guangxi Medical University, Nanning, 530021 China

Retraction of: *Scientific Reports* 10.1038/s41598-019-50471-y, published online 25 September 2019

The Authors have retracted this Article.

After publication concerns were raised with respect to duplication of panels in Figure 4 with those in Figures 2 and 4 of another article by the same authors^1^. When reviewing their data files for this figure the Authors noticed problems with the data presented in other figures in their article but were unable to locate the original data files.

All Authors agree with this retraction.
